# Detection of pathological mechano-acoustic signatures using precision accelerometer contact microphones in patients with pulmonary disorders

**DOI:** 10.1038/s41598-021-92666-2

**Published:** 2021-06-28

**Authors:** Pranav Gupta, Haoran Wen, Lorenzo Di Francesco, Farrokh Ayazi

**Affiliations:** 1 Georgia Institute of Technology, Atlanta, GA 30308 USA; 2 StethX Microsystems, Atlanta, GA 30308 USA; 3 Department of Medicine, Division of General Internal Medicine, Emory University, Atlanta, GA 30303 USA; 4 Ken Byers Professor in Microsystems, Georgia Institute of Technology, Atlanta, GA 30308 USA

**Keywords:** Electrical and electronic engineering, Respiratory signs and symptoms

## Abstract

Monitoring pathological mechano-acoustic signals emanating from the lungs is critical for timely and cost-effective healthcare delivery. Adventitious lung sounds including crackles, wheezes, rhonchi, bronchial breath sounds, stridor or pleural rub and abnormal breathing patterns function as essential clinical biomarkers for the early identification, accurate diagnosis and monitoring of pulmonary disorders. Here, we present a wearable sensor module comprising of a hermetically encapsulated, high precision accelerometer contact microphone (ACM) which enables both episodic and longitudinal assessment of lung sounds, breathing patterns and respiratory rates using a single integrated sensor. This enhanced ACM sensor leverages a nano-gap transduction mechanism to achieve high sensitivity to weak high frequency vibrations occurring on the surface of the skin due to underlying lung pathologies. The performance of the ACM sensor was compared to recordings from a state-of-art digital stethoscope, and the efficacy of the developed system is demonstrated by conducting an exploratory research study aimed at recording pathological mechano-acoustic signals from hospitalized patients with a chronic obstructive pulmonary disease (COPD) exacerbation, pneumonia, and acute decompensated heart failure. This unobtrusive wearable system can enable both episodic and longitudinal evaluation of lung sounds that allow for the early detection and/or ongoing monitoring of pulmonary disease.

## Introduction

The COVID-19 pandemic has highlighted the increasing incidence of acute pulmonary conditions globally, and the lack of adequate healthcare diagnostics for both the early detection and monitoring of life threatening respiratory disorders^1,2^. Pulmonary diseases affect over 1 billion individuals worldwide resulting in premature deaths of 4 million people each year, consequently placing an enormous medical and economic burden on the world healthcare system^1^. The most prevalent of these conditions are chronic obstructive pulmonary disease (COPD), asthma, lower respiratory tract infections (pneumonia and tuberculosis) and lung cancer. A potential effective strategy to lower the morbidity and mortality of these diseases and improve the quality of life of affected individuals is to detect early pre-symptomatic pathophysiological changes such as abnormal breath sounds and/or breathing patterns integral to these conditions. Such a system would allow implementation of earlier disease recognition, diagnosis and management with the hope to slow, alter or cure a patient’s disease expression^3,4^. However, pulmonary diseases such as COPD are universally underdiagnosed and are identified late during a patient’s disease course, often resulting in silent irreparable organ damage^4^.

Episodic and continuous monitoring of mechano-acoustics signals emanating from the lungs using a wearable sensor system can provide valuable diagnostic information by detecting early pathological adventitious breath sounds^5–9^. For instance, “crackles” due to turbulent airflow arising from a bolus of gas passing through airways as they open and close intermittently can be suggestive of a developing pneumonia if acute or the presence of obstructive lung (COPD, bronchiectasis, asthma) if chronic. This finding will have a characteristic signal waveform in the frequency range up to 1 kHz^10,11^. In addition to the high frequency mechano-acoustic lung signals, the macro vibrations corresponding to the chest wall motion and breathing pattern convey additional information regarding the pulmonary health of the patient^12–14^. In patients with congestive heart failure, shallow breathing patterns are indicative of strenuous breathing, and can act as an early biomarker^15,16^. Patients with pneumonia often exhibit a fast respiratory rate (> 30 breaths/min) arising due to both the local lung and systemic inflammatory response, making it a sensitive marker for pneumonia^12,17–19^. Such simple biomarkers provide critical information to diagnose and monitor the clinical deterioration in a patient’s health and can be used for the timely management of many common pulmonary diseases.

Lung mechano-acoustic signals vary over a wide range of intensities and frequencies depending upon the origin of the pathology, and thus require extremely sensitive sensors that can non-invasively capture micro-vibrations occurring on the surface of the skin with high fidelity. Packaging such sensors in a wearable platform can enable episodic and longitudinal monitoring of respiratory conditions and facilitate early detection and comprehensive diagnosis^7,20^. Current diagnostic methods rely mainly on the traditional lung examination using an acoustic stethoscope for the auscultation of the lungs. The relatively short duration of such an auscultatory exam (a few breaths in each area), along with the inability to simultaneously view the respiratory rate and breathing patterns, does not provide for long-term monitoring and comprehensive insight regarding a patient’s respiratory health. Moreover, stethoscopes rely on large membranes to capture the acoustic signals, making them unsuitable to be employed in a wearable system, and are prone to picking up undesired air-borne noise from its surrounding environment^21,22^. Recent works have also focused on development of electrochemical mechano-acoustic sensors for continuous cardiorespiratory monitoring^23^. While these sensors offer conformal ergonomic contact with the skin due to flexible substrates, they are relatively large in size compared to capacitive MEMS devices, have smaller bandwidth and require liquid electrolyte. Miniature high precision accelerometer contact microphones (ACM) have been previously demonstrated to capture heart sounds, respiratory rate, lung sounds, and body motion of an individual simultaneously in a continuous and unobtrusive manner using a single integrated sensor^24,25^.

Here, we present an enhanced ACM sensor that exhibits a wide operational bandwidth (> 10 kHz) along with a low noise performance to accurately capture pathological mechano-acoustic vibrations from the lungs. The hermetically sealed device is packaged into a stand-alone wearable sensor module that can capture and store the vibrational signatures from patients for extended periods of time using a battery-operated data acquisition and storage module. The performance of the wearable sensor is compared to the state-of-art Eko digital stethoscope to assess the feasibility and accuracy of the system in the detection of pathological lung sounds. The efficacy of the system is then demonstrated using an exploratory research study that captures adventitious breath sounds, breathing patterns, and respiratory rate in acutely hospitalized patients with underlying pulmonary conditions and comparing the recorded waveforms to a physician’s clinical observations and recordings from a digital stethoscope.

## Results

### Sensor and system design

**Figure 1:**
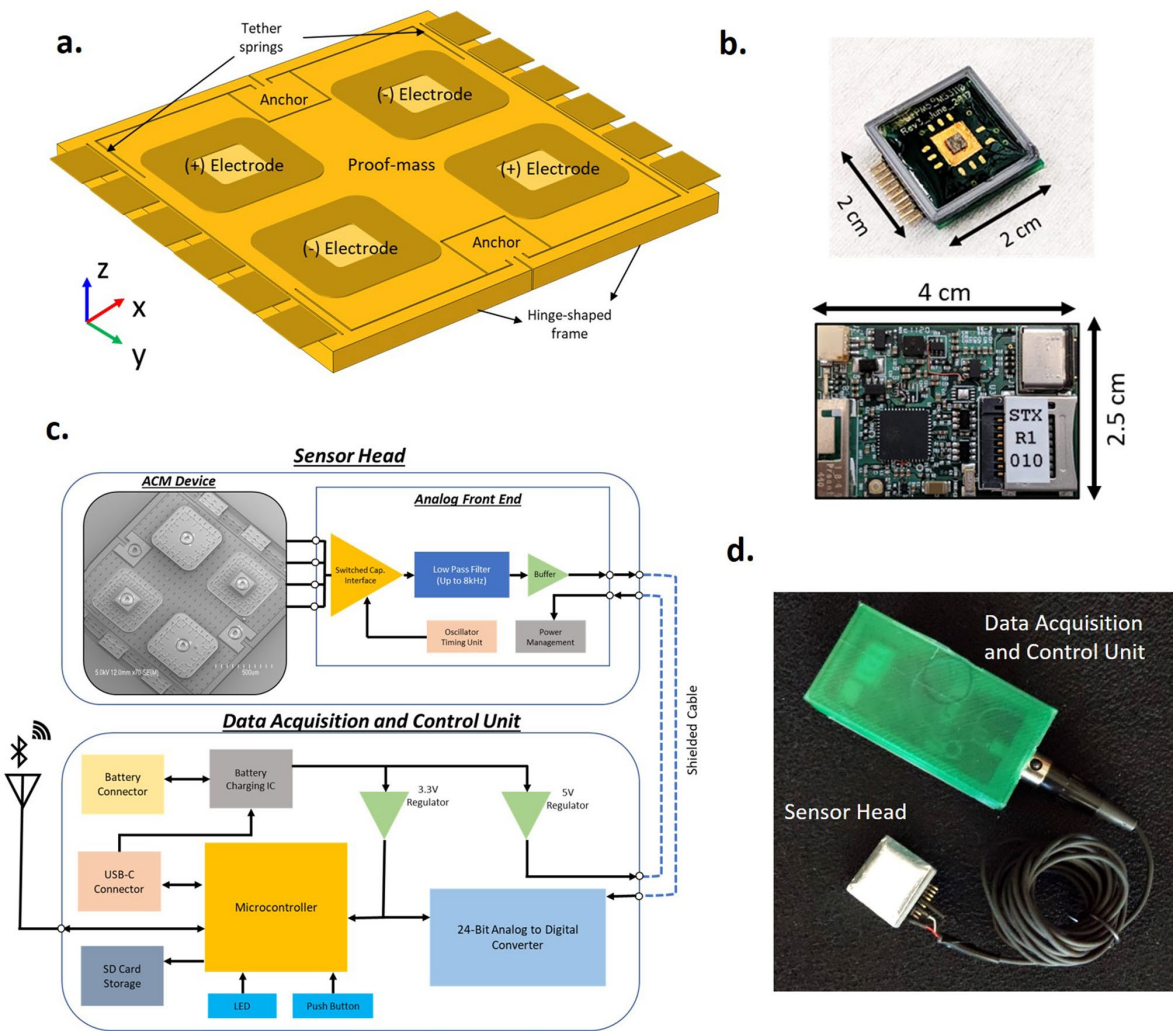
ACM sensor and System. (**a**) Schematic image of ACM design structure with 4 sense electrodes and the proof-mass supported by tether springs at the edges. (**b**) Fabricated PCB boards housing the ACM sensor and interface electronics. The larger control unit board consists of the data aquation and storage components. (**c**) Schematic block diagram of components used in the wearable sensor module. (**d**) image of the packaged wireless sensor system with one ACM sensor head.

The ACM is designed as a translational out-of-plane wideband accelerometer, supported by four springs at the edges of the proof-mass which are located over a center-supported hinge-shaped frame, as shown in Fig. 1(a). Micro-gravity level accelerations occurring due to acoustic vibrations displaces the proof mass perpendicular to plane, resulting in change in capacitance at the sense electrodes. Two pairs of differential sense electrodes are placed within the proof mass area such that the device is not sensitive to angular accelerations and maintains a small form factor. The sensor is designed to operate in vacuum to exhibit ultra-low noise performance (< 10 μg/√Hz) and have a wide operational bandwidth (> 10 kHz), thus providing high sensitivity to micro-gravity level accelerations orthogonal to the device surface.

A battery-operated wearable sensing system comprising of the ACM sensor along with interface electronics, data acquisition unit, wireless communication link and on-board data storage is developed (shown in Fig. 1) for recording mechano-acoustic signals emanating from the body. The system is made up of two parts – the sensor head and the control unit – which are connected using a long flexible shielded cable. This allows the sensor head to be placed at any location in the thoracic region while the control unit may be held on the waist using a simple belt.

The sensor head houses the wire-bonded wafer-level-packaged MEMS devices on a 4-layered miniature circuit board and interfaced with MS3110P– a commercially available off-the-shelf capacitance readout circuit – that exhibits low capacitive resolution (4aF/√Hz) along with a wide bandwidth (programmable up to 8 kHz). The small size of the board reduces the effects of loading, ensures firm contact on conformal skin surface, and prevents any undesired resonant modes that may arise at high frequencies. The sensor head is covered in a thin layer of clear epoxy potting compound DP270 (manufactured by 3 M). The 2-part epoxy (consisting of the base and accelerator) is mixed in a ratio 1:1 by volume and cured at room temperature for 24 h. This epoxy layer protects the bonding wires from damage against handling and allows the MEMS sensor to be placed directly over the testing surface, thus achieving good vibrational coupling.

The control unit contains the electronics responsible for data collection and transmission. The electrical signal coming from the sensor head is digitized using a 24-bit analog-to-digital converter (MAX11270 by Maxim Integrated). The high precision of the data converter is necessary for detection of the weak mechano-acoustic signals. Following Nyquist criterion, a sampling rate of 16,000 samples/second, which is at least twice the maximum signal frequency of 8 kHz is selected to ensure that the recorded signal may be reconstructed for further computer-aided analysis. A microcontroller is used to control the data converter and redirect the digitized signals to either an on-board data storge or transmit it over a Bluetooth link. A push-button is used to switch between these two modes of operation. The system is powered by a 500mAh Li-Po battery, and can be charged directly using the control unit.

### ACM recording of lung signals and comparison to digital stethoscope

**Figure 2:**
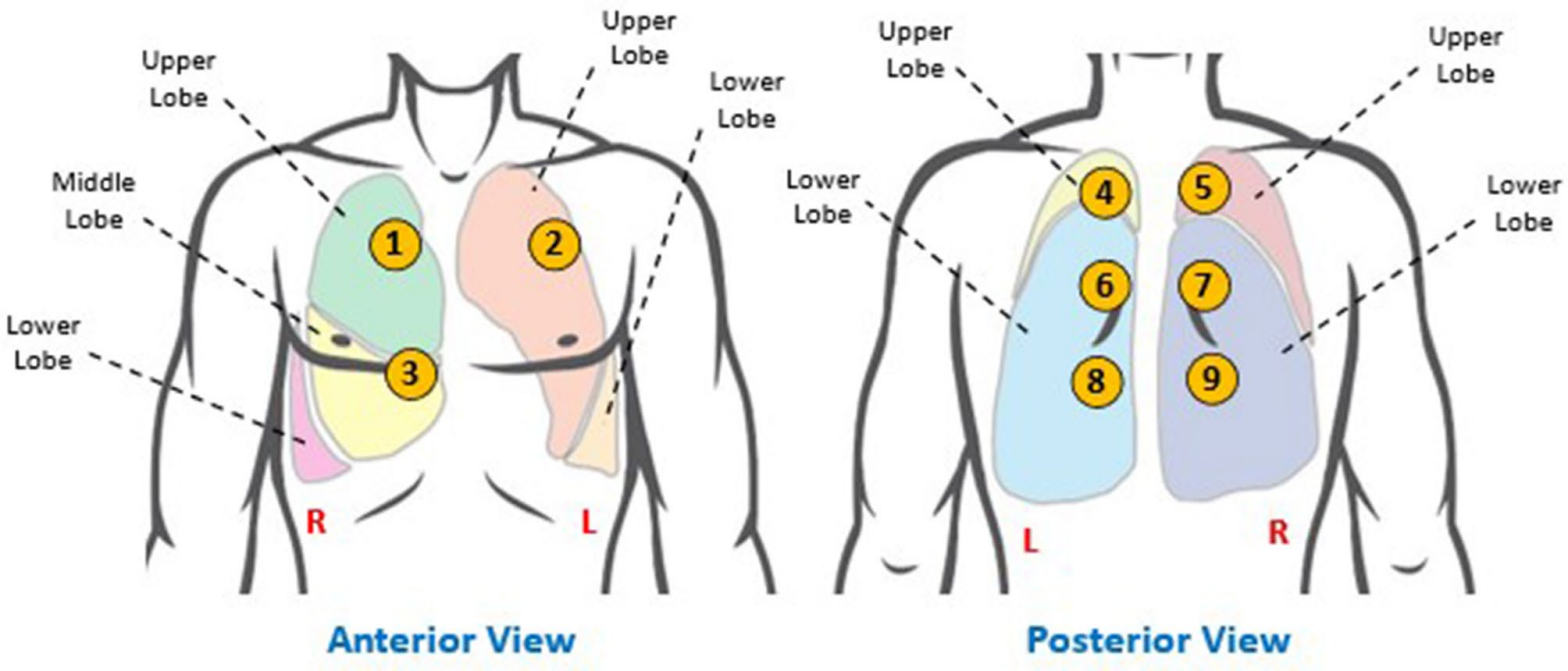
Anatomy of the lungs and auscultation sites. The anterior and posterior view of the body illustrating the location of various lung lobes. Areas numbered 1 through 9 indicate the auscultation sites used for data recording from patients. The locations are chosen to examine majority of the lung area during the study.

The lungs occupy the major volume of the thoracic cavity and are responsible for the oxygenation of the blood and are each anatomically composed of smaller segment called lobes^26^. The right lung is made up of three lobes: the upper, middle, and lower lobes, while the left lung consists of two lobes: the upper and lower lobes, as shown in Fig. 2. During inhalation, the air accesses the lungs via the trachea which branches out into the bronchi ultimately reaching distally the alveolar air sacs of the lungs. These alveoli present inside the lungs are responsible for the absorption of the oxygen-rich air into the bloodstream.

The characteristic features of the air flow are modulated as it passes through these pulmonary structures, resulting in the production of normal breath sounds^10,27^. The motional pattern of the airflow determines the characteristic features of the breath sounds. A laminar flow – that is parallel to the walls of the airways – results in low frequency and silent vibration, while a turbulent flow – characterized by disorganized and chaotic motion – produces high frequency audible sounds during respiration. The outer lining of the lungs and chest wall act as a low-pass filter, not allowing high frequency sounds to pass through. The sounds heard over the chest wall contain signals ranging from 100 Hz to 5 kHz^11,27^.

Due to the large anatomical size of the lungs, multiple auscultation locations must be chosen to capture high-fidelity signals occurring from the 5 major lobes of the lungs. For this study, nine auscultation locations that mimic real-time lung auscultation were utilized: – three areas on the anterior and six areas on the posterior side – to capture a comprehensive analysis of the lung sounds in each subject. The auscultation sites were chosen such that the signal quality was not compromised by the heart sounds, and each area may be easily accessed for the sensor placement. These auscultation locations are anatomically in Fig. 2.

To assess the feasibility of using the developed sensor module to detect pathological lung sounds, the performance of the sensor was first qualitatively compared against the recorded lung sounds from the Eko digital stethoscope. Lung sound data was recorded from patients using both systems, and a time-domain comparison is conducted to identify the similarity between the two recorded waveforms. The sensor was attached to the skin using medical-grade adhesive tape to ensure a firm and secure contact between the sensor surface and the skin. The control unit of the sensor module was held by the physician to begin and end the data recording session. The patient was seated in an upright position and instructed to perform a series of deep breaths. Both systems were placed one-by-one at the 9 auscultation sites and data was recorded over a continuous 20-s period at each position.

**Figure 3:**
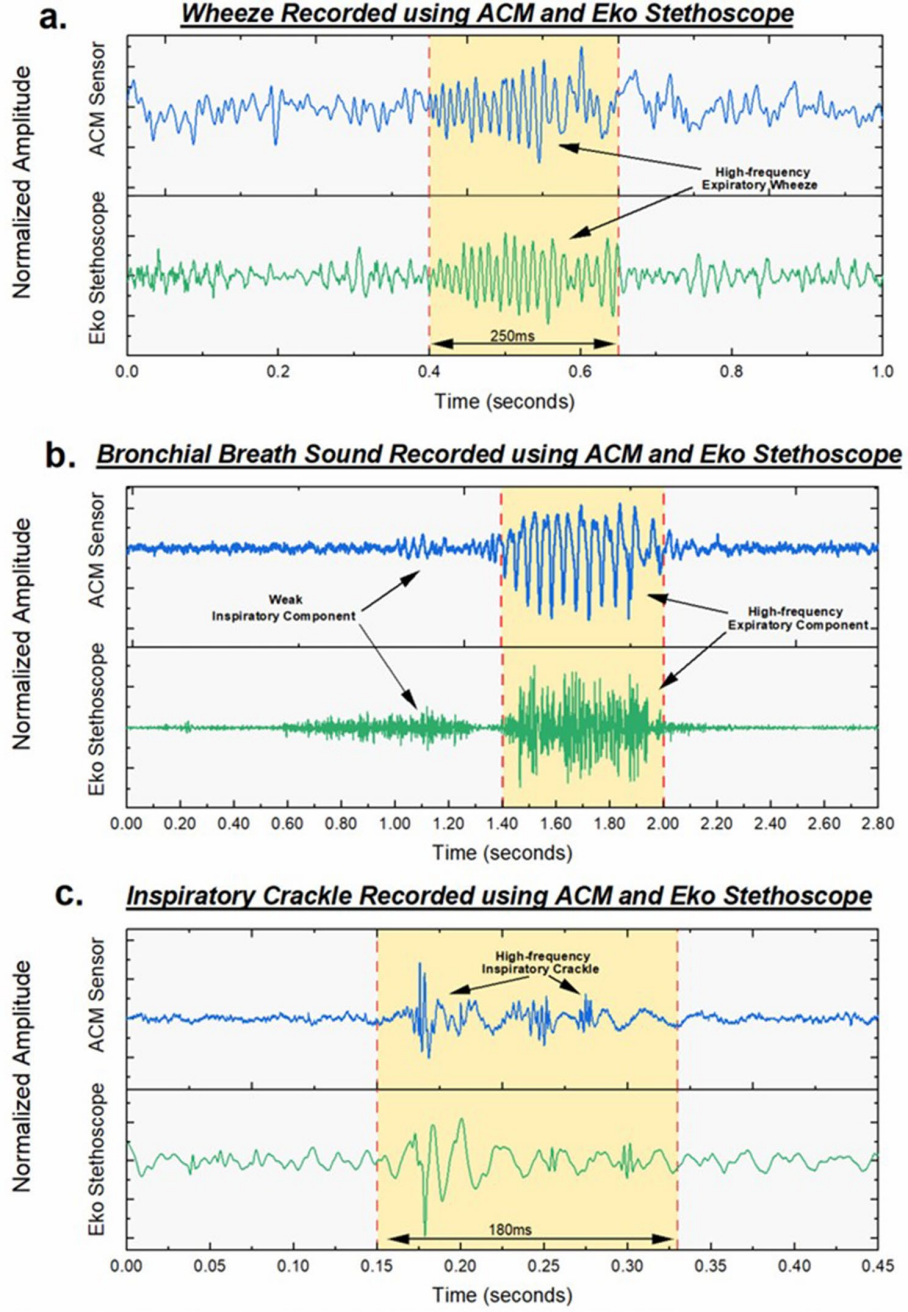
Comparison of ACM performance with Eko digital stethoscope. (**a**) Signal waveforms of expiratory wheeze captured using ACM sensor and digital stethoscope indicating a time duration of 250 ms. Signal exhibits sinusoidal characteristic. (**b**)Signal waveforms of expiratory bronchial breath sounds exhibiting loud, high frequency signals. **c** Signal waveforms of inspiratory crackle exhibiting short bursts of high frequency signals with a total duration of 180 ms.

Wheezes, which are musical high-pitched sounds, can often be heard during both inspiration and/or more commonly during expiration. They exhibit a sinusoidal characteristic occurring in a frequency range of 100 Hz–5 kHz, with a typical duration more than 80ms^11,28^. They are associated with airway narrowing and are commonly present in patients with asthma or COPD exacerbations. Figure 3(a) shows typical recordings of wheezing waveform identified during the expiration phase using the ACM sensor and the Eko digital stethoscope from a patient hospitalized with a COPD exacerbation. Both waveforms exhibit a high degree of similarity, with sinusoidal patterns occurring for a duration of 250 ms.

Bronchial breath sounds are loud, hollow, and high-pitched sounds occurring commonly due to lung consolidation. They exhibit a longer expiratory phase as compared to the inspiratory phase and have a distinct pause between the two phases^11,29^. Presence of bronchial breath sounds is an early indicator of developing pneumonia. Figure 3(b) shows the recorded bronchial breath sounds from a patient with diagnosed with pneumonia. The waveforms showcase a comparison of recordings of the Eko stethoscope and the ACM sensor in capturing the weak inspiratory and strong expiratory components of the adventitious breath sound. Both signals exhibit a similar shape and envelope pattern but may not exactly correspond to similar frequency content. This can arise due to imperfect contact of the ACM sensor with the skin surface, causing the sensor to capture only high-amplitude low-frequency content accurately. Moreover, since the data collection was done serially, the two recorded signals are not identical as they were recorded at two separate times, and hence may show different frequency content. Signal quality can be enhanced by developing improved sensor-skin interface and reducing the noise of sensor interface electronics.

Crackles (either fine or coarse) are short explosive sounds, normally heard during the inspiration phase. These are general non-musical in nature, and have sharp high-frequency features, with a typical frequency less than a kilohertz (350–640 Hz). Presence of these sounds is one of the earliest biomarkers in identification of diseases such as obstructive lung disease (COPD, asthma)^10^, acute decompensated heart failure^30^, pneumonia^17^ and interstitial lung disease (which may be identified by the presence of fine crackles)^31^. Accurate assessment of crackles is helpful since they may act as an early biomarker and can be present before detection of the disease by chest radiography^32^. Figure 3(c) shows the recorded waveforms of crackles occurring during the inspiratory phase in a patient with acute decompensated heart failure. Both waveforms showcase short duration signals with high-frequency content, occurring for a total of 180 ms. The ACM signal however shows a richer signal quality due to its very high sensitivity to weak, high frequency vibrations.

In addition to recording auscultatory data, it is potentially beneficial for physicians to also visualize the breathing pattern/s and correlative respiratory rate of their patients for the early identification of developing pulmonary diseases. A shallow breathing pattern with a high respiratory rate can suggest shortness of breath potentially due to underlying lung pathology. The ACM sensor, being sensitive to vibrations ranging from DC up to several kilohertz is sensitive to the motions of the chest wall, and captures the respiratory rate and breathing patterns with high-fidelity, thereby enhancing the information available to physicians for a comprehensive diagnosis^25^. Supplementary Fig. 7 showcases different breathing patterns corresponding to deep, normal, and shallow breathing.

### Patient data

An exploratory research study to evaluate the feasibility of the ACM device in the identification of lung pathologies was conducted, and involved patients hospitalized at the Grady Memorial Hospital in Atlanta with varying degrees of pulmonary disease. The study was aimed at recording auscultatory data and breathing pattern waveforms using the ACM device and associating the waveforms with characteristic features of the patient’s underlying condition. Three patients – each admitted with a COPD exacerbation, acute decompensated heart failure, and pneumonia – were chosen for the comparative study. The patients had body mass indexes (BMIs) ranging between 22.7–27 and were all male in the age group of above 40 years old. A protocol involving data collection from multiple auscultation locations using the ACM sensor and Eko stethoscope while seated in an upright position was employed. A physician recorded the clinical observations based on traditional auscultation using the Eko digital stethoscope, and those recordings were compared to the anatomically matched recorded ACM signal. The physician was blinded to the recorded ACM signal, and made an assessment based on auditory perception of the Eko stethoscope signals only.

**Figure 4:**
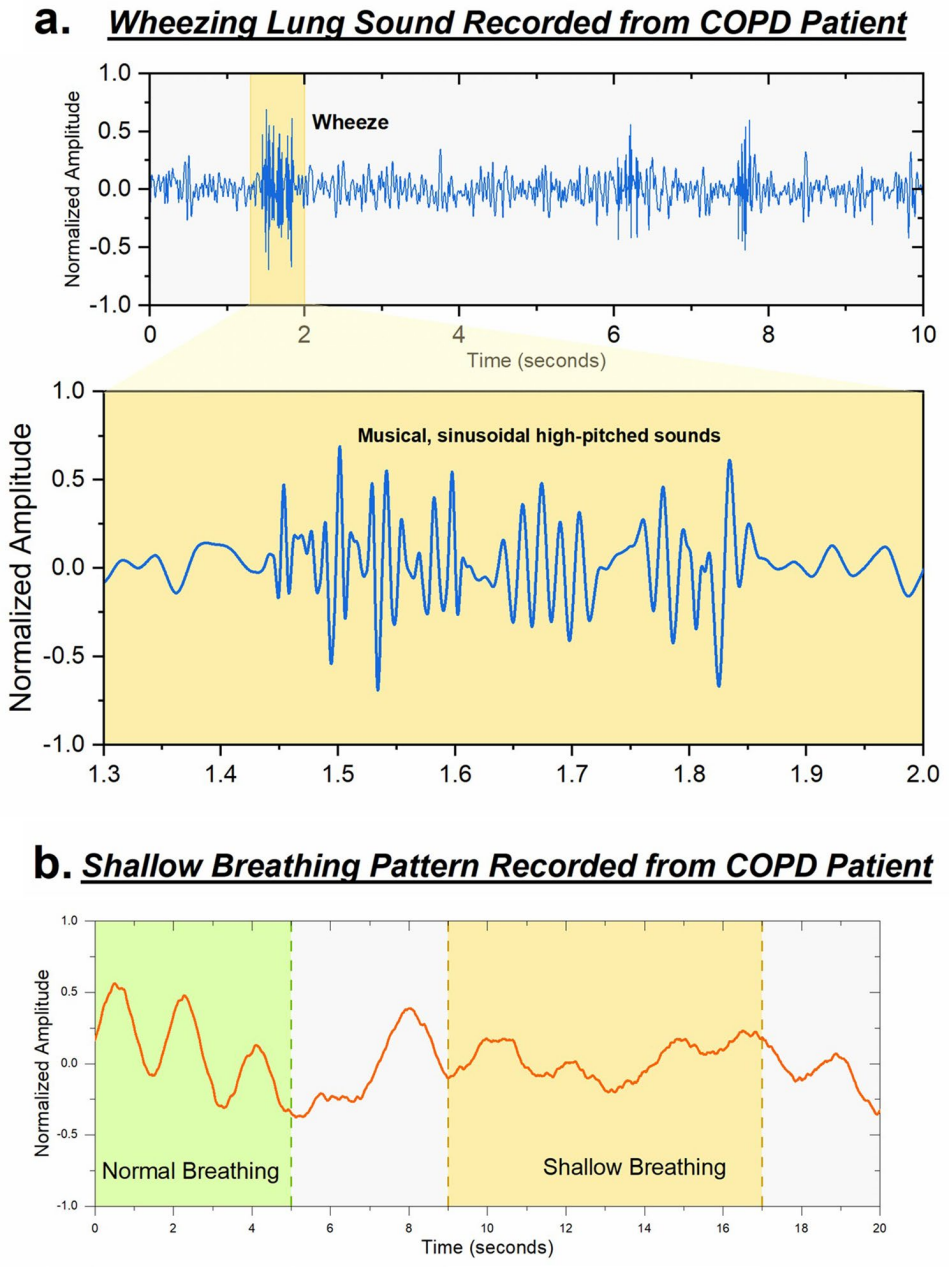
Pathological signals recorded from COPD patient. (**a**) Wheezing lung sound recorded from left upper lobe on the posterior side from patient with COPD. Wheezing periods are highlighted in the waveform. (**b**) COPD patient exhibiting difficulty in breathing, characterized by a shallow breathing pattern.

Figure 4(a) shows the recorded pathological lungs sound of wheezing recorded from a patient with a COPD exacerbation. The representative data was recorded from the left upper lobe on the posterior side. In the case of severe airway obstruction, the airflow may be minimal leading to absence of clinical wheezing. During this situation, it is beneficial to also be monitoring the respiratory rate and breathing pattern of such individuals which may be noted to exhibit significant respiratory distress with an abnormally high respiratory rate with decreased breath sounds and the absence of wheezing. Figure 4(b) shows an abnormal breathing pattern of the same patient, exhibiting a series of shallow rapid breaths. This indicates acute labored breathing and can be an additional major biomarker for a severe COPD exacerbation^4^.

**Figure 5:**
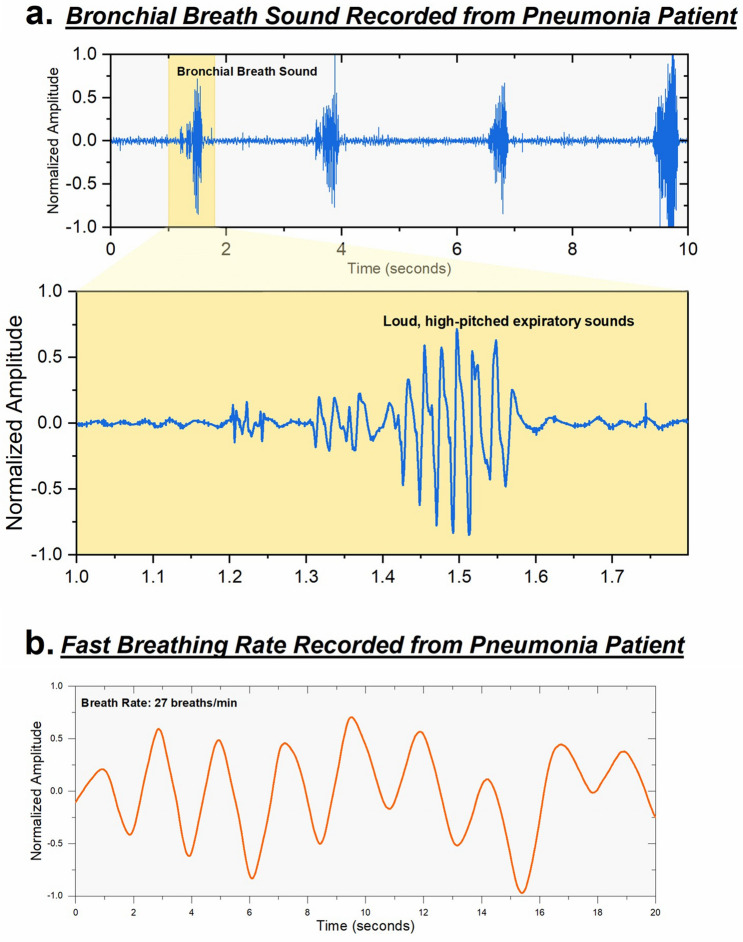
Pathological signals recorded from pneumonia patient. (**a**) Bronchial breath sound recorded from right inferior lobe on the posterior side from patient with pneumonia. Presence of these sounds at peripheral airways in indicative of the disease. (**b**) Pneumonia patient exhibiting a fast-paced breath rate (27 breaths/min) with a normal breathing pattern.

Another commonly occurring respiratory disorder is pneumonia, which is characterized by pathologic fluid accumulation in the lungs leading to generation of crackles upon auscultation. In addition to the localized crackles in pneumonia, the presence of another adventitial lung sound: bronchial breath sounds found on auscultation is indicative of the development of pneumonia that has formed lung consolidation^33^. As shown in Fig. 5(a), noted bronchial breath sounds were detected in this patient hospitalized with pneumonia. The representative sounds were identified at the right lower lobe on the posterior side corresponding area of pneumonia identified on chest radiography. These loud, tubular sounds were observed during the exhalation phase in this patient. In addition to the presence of localized bronchial breath sounds, the patient’s fast respiratory rate (~ 27 breaths/min) of Fig. 5(b) has additional clinical significance in patients with pneumonia^18^.

**Figure 6:**
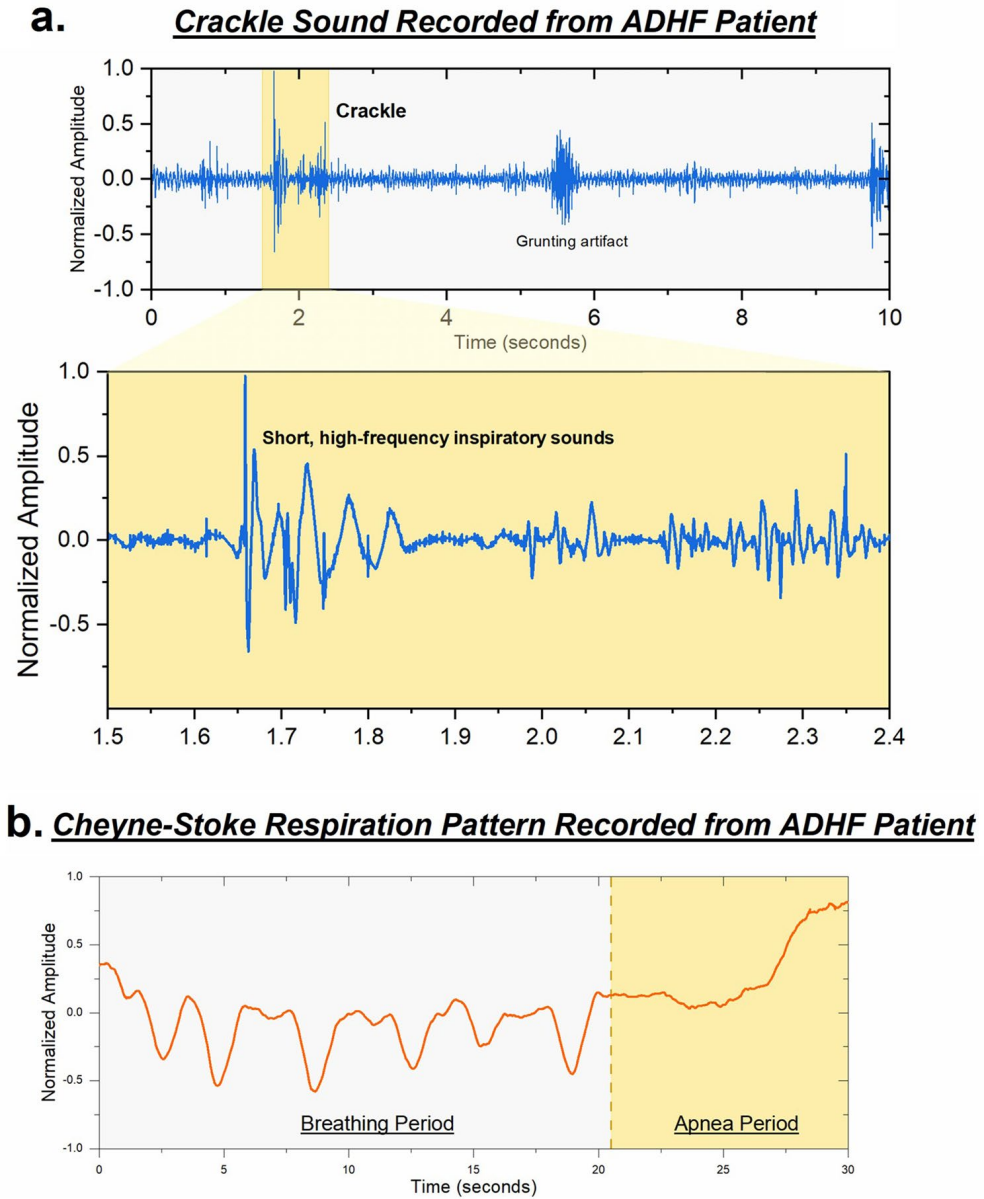
Pathological signals recorded from ADHF patient. (**a**) Presence of high frequency inspiratory crackle at lower lobes of the lungs indicating fluid accumulation. (**b**) Cheyne-Stokes respiration (CSR) pattern recorded from patient with ADHF and is characterized by a period of breathing followed by a period of apnea. CSR is a common indicator of heart failure.

Acute decompensated heart failure (ADHF) is a cardiopulmonary disorder with a high prevalence where disease of the heart leads to fluid accumulation in the lungs^16,34^. Presence of bilateral crackles notably at the bases of the lungs is a strong indicator of potential decompensated heart failure. Figure 6(a) shows the ACM data recorded from such a patient, exhibiting crackles and high frequency signals at the lower lobes on the posterior side. Vibrational signatures corresponding to grunting during the respiration process are also observed, indicating discomfort during breathing. In addition to the presence of crackles and grunting artifacts, a characteristic breathing pattern, namely the Cheyne-Stokes respiration was observed in this patient, which is often noted in patients with advanced decompensated heart failure^35^. This pathognomonic pattern consists of a cyclical series of fast breaths followed by a period of apnea. The respiration pattern recorded for this patient is shown in Fig. 6(b). The biomarkers of abnormal adventitial sounds of bilateral lower lobe crackles with noted Cheyne Stokes respiratory breathing pattern provide a synergistic indication of the presence of decompensated heart failure.

## Discussions

Early detection of pathological signatures in mechano-acoustic signals emanating from the lungs is critical to diagnose, monitor and manage pulmonary disorders and improve the quality of patient care. Our miniature ACM sensor is packaged into a wearable sensor module that can record data episodically and longitudinally to enhance the diagnosis of pulmonary disorders by analyzing the detected abnormal lung signals in the early and late stages of the disease. The sensor is responsive to a wide band of vibrational frequencies, while also being sensitive to micro-movements of the chest wall with high precision. The ACM sensor module can be operated as a wearable stand-alone, wireless system, not requiring any external cables or wireless link for data recording. The low power sensor and electronics ensure a long battery life for the system, along with a high storage capacity of up to 7000 min of recording while using a 32 GB SD card.

In comparison to other works^36–38^ in this area, the ACM sensor offers several advantages due to its very small footprint, and high vibration sensitivity in a broad frequency band extending all the way to DC. The ACM sensor leverages these qualities to detect weak mechano-acoustic vibrations emanating from the lungs, which typically requires bulky stethoscopes, while being able to simultaneously detect breathing rate and body position using the same hermetically-sealed small device.

The modules offers a flexible system to record the lung sounds along with the breathing patterns and respiratory rate of the individual – a feature that is currently unavailable in the state-of-art digital stethoscopes. This allows physicians to locate abnormal lung sounds with respect to the respiratory cycle, providing a richer information set to perform a comprehensive diagnosis. This information may be further augmented by employing the system intermittently in an ambulatory setting to listen to lung sounds as the body is put under normal daily stresses. However, due to an overlap in frequency ranges for certain respiratory patterns and body motion, advanced signal processing techniques are required to isolate the signal features and extract the useful information for diagnostic purposes. Simultaneous use of multiple ACM sensors embedded in a lung harness can be conducted to record the mechano-acoustic signals from multiple auscultation points and applying signal processing techniques such as decomposition^39^, and independent component analysis^40^ to reduce motion artifacts could allow a more rapid and comprehensive pulmonary assessment.

All data recorded from the patients during the research study were conducted under silent and sedentary conditions to minimize the effects of motion and vocal artifacts. All patient data was collected within the first 24 h of hospitalization, thus providing a superior symptomatic signal. However, the high sensitivity of the ACM sensor can be leveraged for earlier detection of these common pulmonary diseases and therefore has the theoretical potential to improve the quality of patient care thru earlier diagnosis and faster therapeutic management.

In summary, we presented a miniature wearable sensor system which can record lung sounds and respiratory rate with high-fidelity for both episodic and long-term monitoring of patients in an unobtrusive manner. This study was conducted on patients with preexisting acute pulmonary conditions to monitor the pathological mechano-acoustic signals emanating from the lungs by placing the sensor in the thoracic region at multiple auscultation locations and recording characteristic waveforms corresponding to the underlying condition. This study opens the possibility for earlier detection and therapeutic intervention of respiratory diseases that has the potential to lead to better patient and healthcare outcomes.

## Materials and methods

### ACM fabrication process

The ACM is fabricated using the HARPSS + process^41,42^, which is a combination of LPCVD and DRIE processes, with the transduction nano-gaps defined by thermal oxidation of silicon, as shown in supplementary Fig. 1. The process starts with a silicon on insulator (SOI) wafer comprising of a 40 µm device layer and a 500 µm handle layer, separated an oxide layer of 2 µm thickness. The device layer is thermally oxidized to form a hard mask and is followed by patterning of the top surface. Using DRIE techniques, trenches are etched in the device layer that define the structure of the sensor. The DRIE trenches are then filled using LPCVD tetraethyl orthosilicate (TEOS) through a process of repeated conformal deposition and top surface etch back. The top surface is then patterned, which exposes the regions for transduction and electrode anchoring. The exposed region is thermally oxidized to form the top sacrificial oxide layer (250 nm in thickness) for the sense electrodes. Finally, polysilicon is deposited and patterned to for the sense electrode. The devices are released in an HF solution. The capping wafer is created using a simple silicon wafer consisting of deep polysilicon pillars with oxide isolation to create Through-silicon vias (TSV), which are used to create an external connection path from the capping wafer to the sense electrodes. The cavity etched inside the capping wafer is depth-controlled to ensure the desired package pressure level. Using eutectic bonding, the capping wafer is fixed to the base wafer consisting of the fabricated device. Finally, the TSVs are exposed by grinding the excess capping silicon wafer capping wafer to thickness of 300 µm. A PECVD oxide and metal electroplating process is employed to form the electrical routing on the packaged device.

### System characterization

The fabricated devices are tested for performance using standard shaker table tests. The sensor demonstrates a sensitivity of 271 mV/g and a linear response up to ± 4 g acceleration level. A cross axis sensitivity of less than 3% is measured, occurring mainly due to misalignments of the sensor to the measurement setup. The low noise performance of the microsensor is presented in Supplementary Fig. 2, exhibiting a velocity random walk of 55 μg/√Hz, which is primarily limited by the interfacing electronics. The Brownian noise floor of the MEMS device is < 10 μg/√Hz making it an ideal candidate for sensing weak mechano-acoustic signals. The operational bandwidth of the device is measured by applying sinusoidal acceleration at varying frequencies using the shaker table, and the sensor response is characterized as shown in Supplementary Fig. 2. The flat response demonstrates the sensor’s ability to measure accelerations up to 10 kHz frequency with high fidelity. The complete specifications of the sensor are listed in Supplementary Table 1.

The fabricated capacitive transducer is interfaced with a capacitance readout circuit (off-the-shelf electronics – MS3110 by Irvine Sensors) on a miniature evaluation board electronic board with the size of 2 cm × 2 cm. The sensor board is connected to the control unit consisting of the data acquisition circuit along with other peripheral components such as the storage SD card and battery. To measure sensitivity, the sensor board is mounted on the shaker table (ET-126HF by LabWorks Inc.) and a sinusoidal 1 g acceleration is applied at 1 kHz frequency. Using the 35760A Dynamic Signal Analyzer a measurement of the device scale factor is conducted, showing 271 mV/g. The operational bandwidth of the device was measured by ramping up the excitation frequency on the shaker table up to 10 kHz (limit of the shaker table), and a flat sensor response was observed, confirming high operational bandwidth.

A control unit for the sensor module packaged in a 3D printed housing, giving easy access to buttons, SD card slot and the battery charging port. The system is designed to be light-weight and portable to enable use of the ACM in a wearable form-factor. Medical-grade adhesive tape (manufactured by 3 M) are used to hold the sensor in place over the skin while minimizing any irritation and suppressing the undesired rubbing noise that may arise due to motion. Only a small sensor area is in contact with skin and this device did not cause any burden for the wearers. The data acquisition system records the sensor output with sampling rate of 16 k samples/sec and stores it on the mounted SD card in binary format. The data is transferred from the SD card onto a computer and converted into a numerically readable format. Data processing and filtering techniques are employed using a MATLAB program to separate the low frequency component of the chest wall motion from the high frequency audible components. The breathing pattern and respiratory signals corresponding to movements of the body at low frequencies is separated by a low pass filter with cutoff frequency 20 Hz along with a moving mean filter with a window size of 1 s. The lung sounds occurring at a higher frequency range are obtained by applying a high pass to eliminate frequency components below 20 Hz followed by a wavelet denoising algorithm.

### Exploratory research study

All the human subjects participated voluntary with informed consent. The protocol was approved by Emory University and Georgia Institute of Technology Institutional Review Board (IRB# IRB00105563). The process of data collection from patients was supervised by an experienced and authorized physician, and the sensors were studied on patients in Grady Memorial Hospital in Atlanta. All methods were carried out in accordance with the relevant guidelines and regulations of approved by the review board.

## Supplementary Information

Supplementary Information.

## Data Availability

All recorded datasets will be available to any investigator upon reasonable request.

## Code availability

The code will be available to any investigator upon reasonable request.
