# Supplementary material for: Detection of pathological mechano-acoustic signatures using precision accelerometer contact microphones in patients with pulmonary disorders
Source: Sci Rep. 2021 Jun 28;11:13427. doi: 10.1038/s41598-021-92666-2 (PMC8238985; doi:10.1038/s41598-021-92666-2)
Supplement: Supplementary file 1 — Supplementary Information. [file 41598_2021_92666_MOESM1_ESM.pdf]

Detection of Pathological Mechano-Acoustic Signatures Using Precision Accelerometer Contact Microphones in Patients with Pulmonary Disorders

*Pranav Gupta**
Georgia Institute of Technology, Atlanta, GA 30308, USA
Email: pgupta97@gatech.edu

Haoran Wen
StethX Microsystems, Atlanta, GA 30308, USA
Email: haoran@stethx.com

Lorenzo Di Francesco, MD
Professor of Medicine
Department of Medicine
Division of General Internal Medicine
Emory University, Atlanta, GA 30303, USA
Email: ldifran@emory.edu

*Farrokh Ayazi**
Ken Byers Professor in Microsystems
Georgia Institute of Technology, Atlanta, GA 30308, USA
Email: ayazi@ gatech.edu

***** Corresponding Authors

Supplementary Notes

Improvements to ACM Performance

To enable sensitivity to pathological lung sounds which are significantly lower in amplitude as compared to cardiac vibrations, the designed ACM sensor must offer high resolution along with a wider bandwidth. The operational bandwidth of the ACM is primarily determined by the resonant frequency of the MEMS structure and the air damping caused by the sub-micron capacitive gaps. To manage air damping and minimize the environmental effects on the sensors, the ACM is designed to be wafer level packaged into a 2.8 mm × 2.8 mm hermetically encapsulated package with an internal pressure level of 10 Torr. The ACM has a resonant frequency of 14.3 kHz, to capture a wide range of pathological acoustic vibrations from the lungs. In comparison to our previous ACM design, the enhanced performance of the presented ACM sensor is achieved by reducing the capacitive transduction gap size from 270nm to 250nm, while advancing the designed structure for a more robust functionality. The sensor topology is switched from a cantilever to a translational mode of operation to lower the mechanical noise floor and reduce the device sensitivity to angular accelerations.

Here, the ACM is designed as a translational out-of-plane accelerometer, supported by four springs at the edges of the proof mass. The springs are located over a center-supported hinge-shaped frame, responding to accelerations perpendicular to the surface. Upon externally applied acceleration, the proof-mass moves translationally perpendicular to the plane, while the hinge-shaped frame rotates about the y-axis. The edges of the frame constitute the damping electrodes and shock-stop structures which are displaced greater than the proof-mass, thus causing additional damping under normal operation and preventing damage to the device under high-g shock conditions which may occur for example, when a person using the ACM falls.

Supplementary Figures

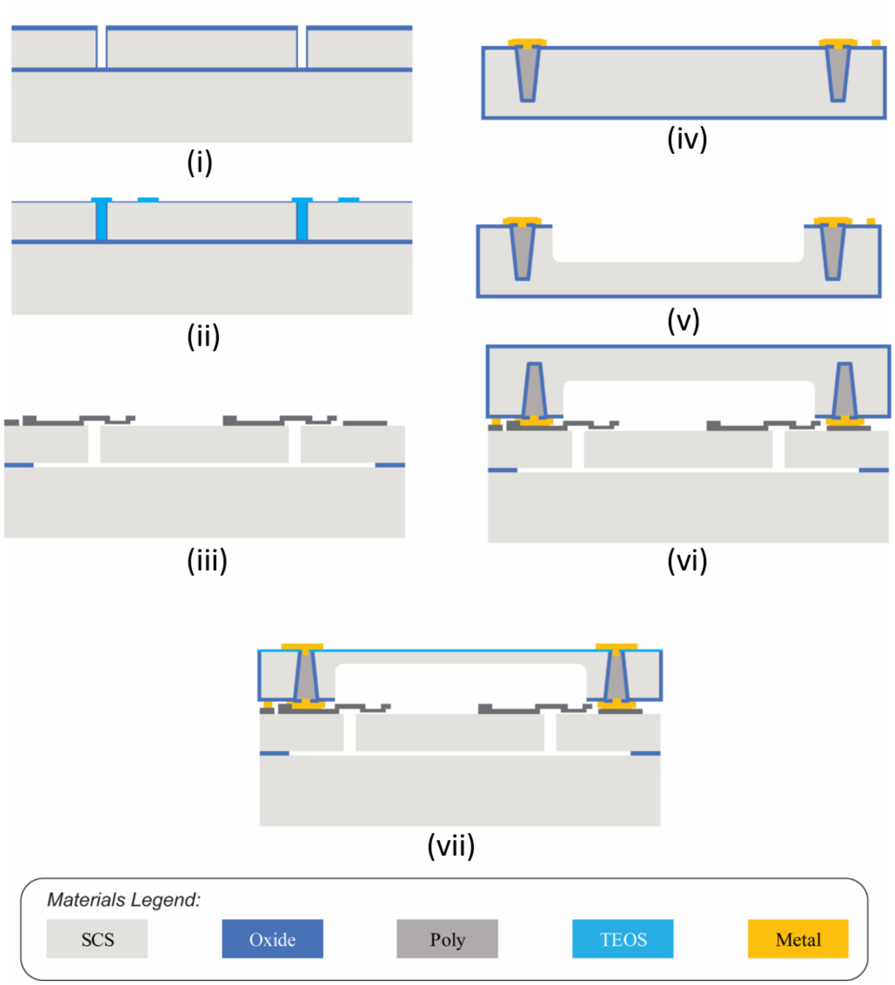

Supplementary Figure 1 | Cross-sectional view illustrating step-by-step fabrication of the ACM. The sensor is fabricated in two parts – the device wafer and the capping wafer – which are fused together using eutectic bonding the final fabrication step. The device is fabricated using the HARPSS+ process which enables implementation of nano-scale transduction gaps. The capping wafer consists of through-silicon-vias (TSV) which create an electrical connection to the sensing electrodes after fabrication.

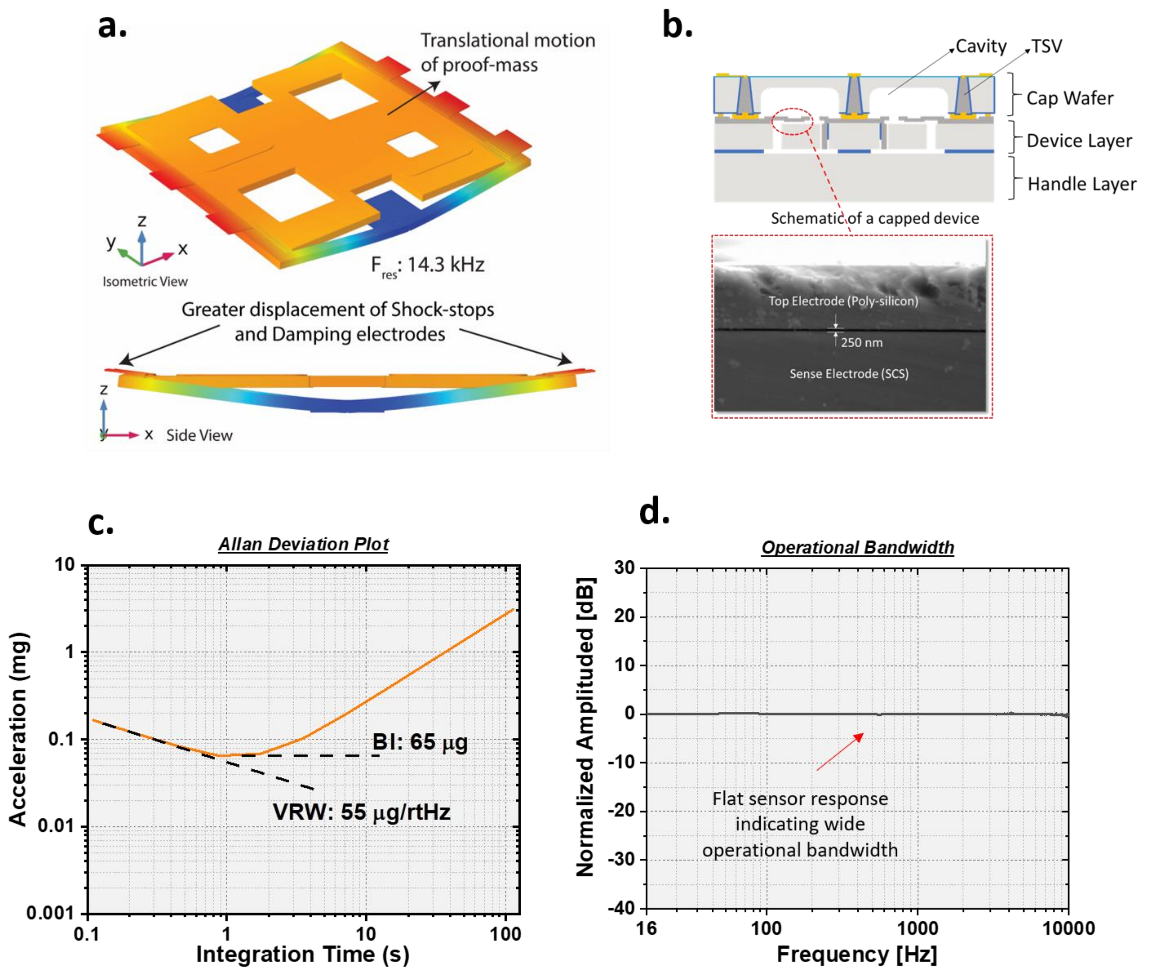

Supplementary Figure 2 | ACM Sensor design and performance characterization. a. simulated resonant mode shape of the sensor showcasing the translational motion of the proof-mass, **b.** cross sectional schematic view of the fabricated senor with the SEM image of the nano-scale transduction gap **c.** Allan deviation plot exhibiting low noise performance of 55 µg/√Hz **d.** transducer response to normally applied acceleration with frequency varying up to 10kHz. Flat response indicates wide operational bandwidth of the sensor.

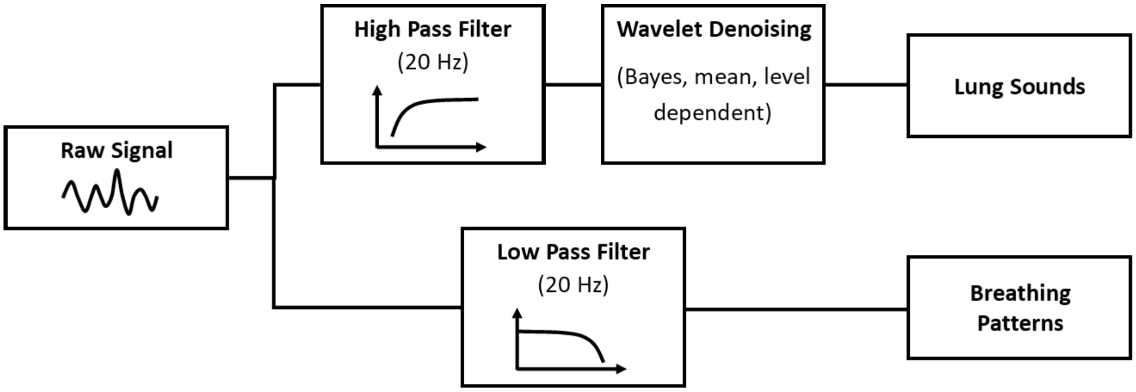

Supplementary Figure 3 | Signal Processing Flow. The raw ACM signal is filtered using a low pass IIR filter (<20Hz) to obtain the low frequency breathing pattern signals. High pass filtering (>20Hz) followed by wavelet denoising is applied to obtain high-fidelity lung sound signals.

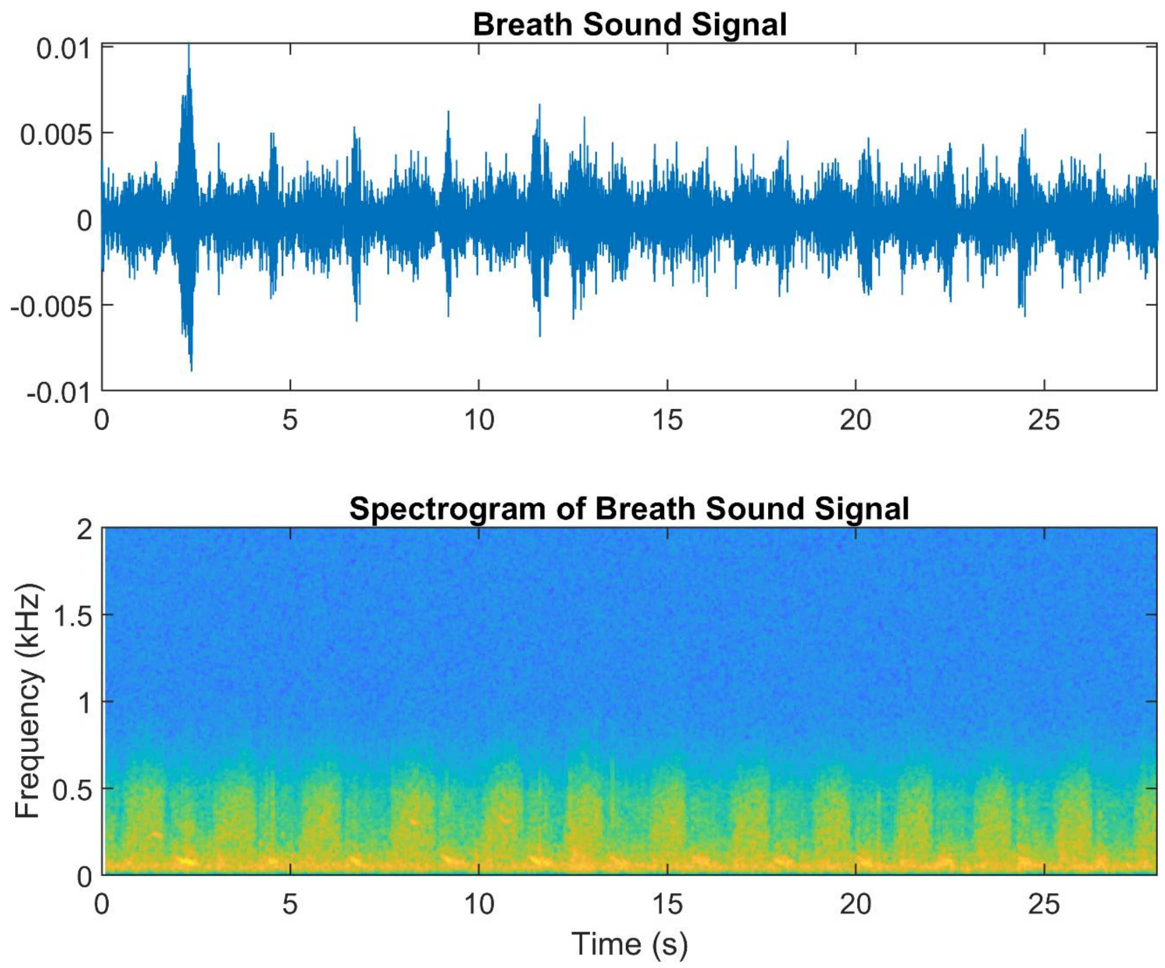

Supplementary Figure 4 | Lung sounds recorded from COPD patient using Eko stethoscope. Wheezing lung sounds recorded from patient with COPD from the left upper lobe on the posterior side. The spectrogram showcases the high frequency content occurring with each breath. Due to absence of low frequency signal, shallow breathing pattern is not seen using the Eko stethoscope.

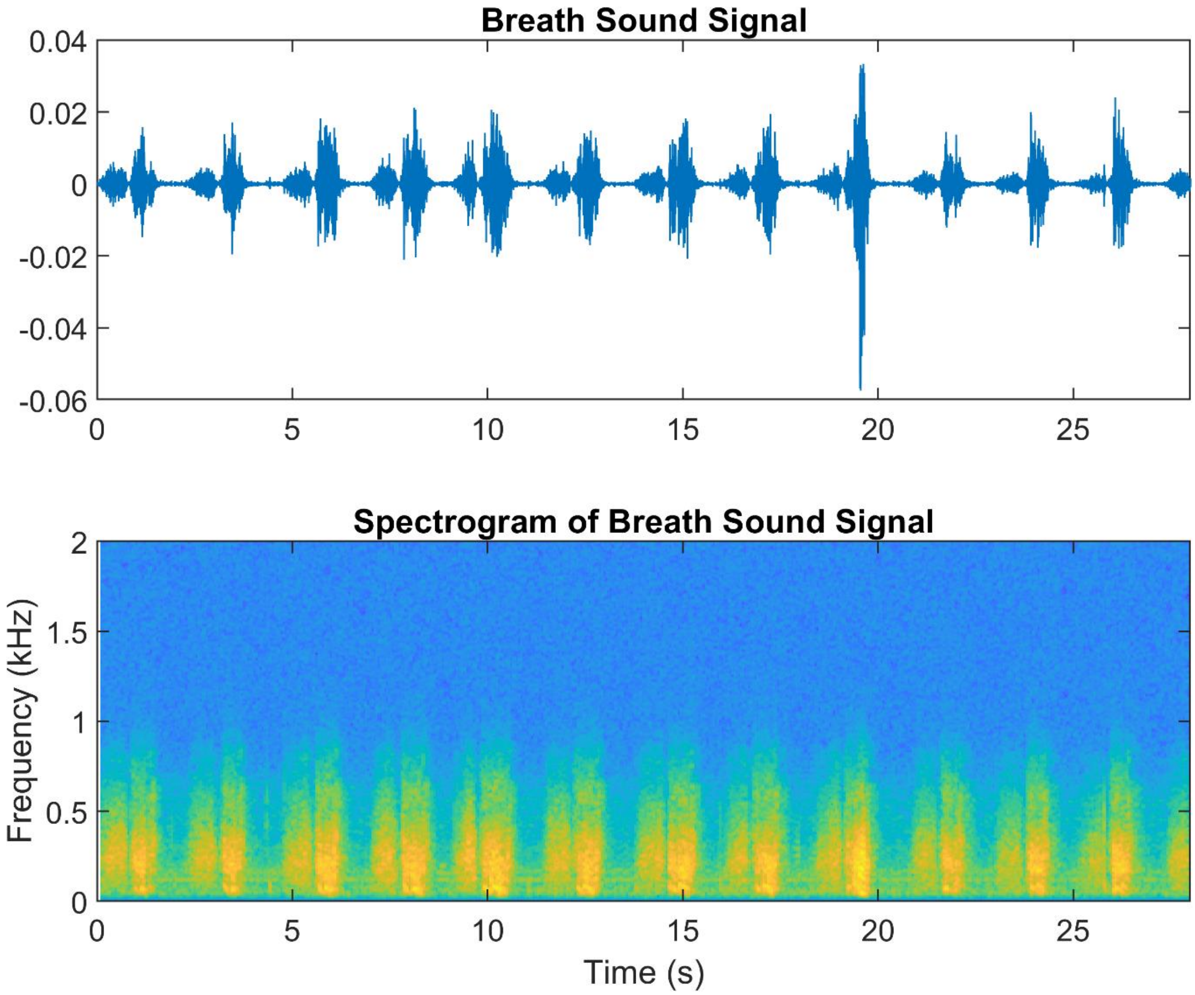

Supplementary Figure 5 | Lung sounds recorded from pneumonia patient using Eko stethoscope. Bronchial breath sounds recorded from patient with pneumonia from the peripheral airways in the right inferior lobe on the posterior side. The breathing rate may be computed using the periodicity of the signal.

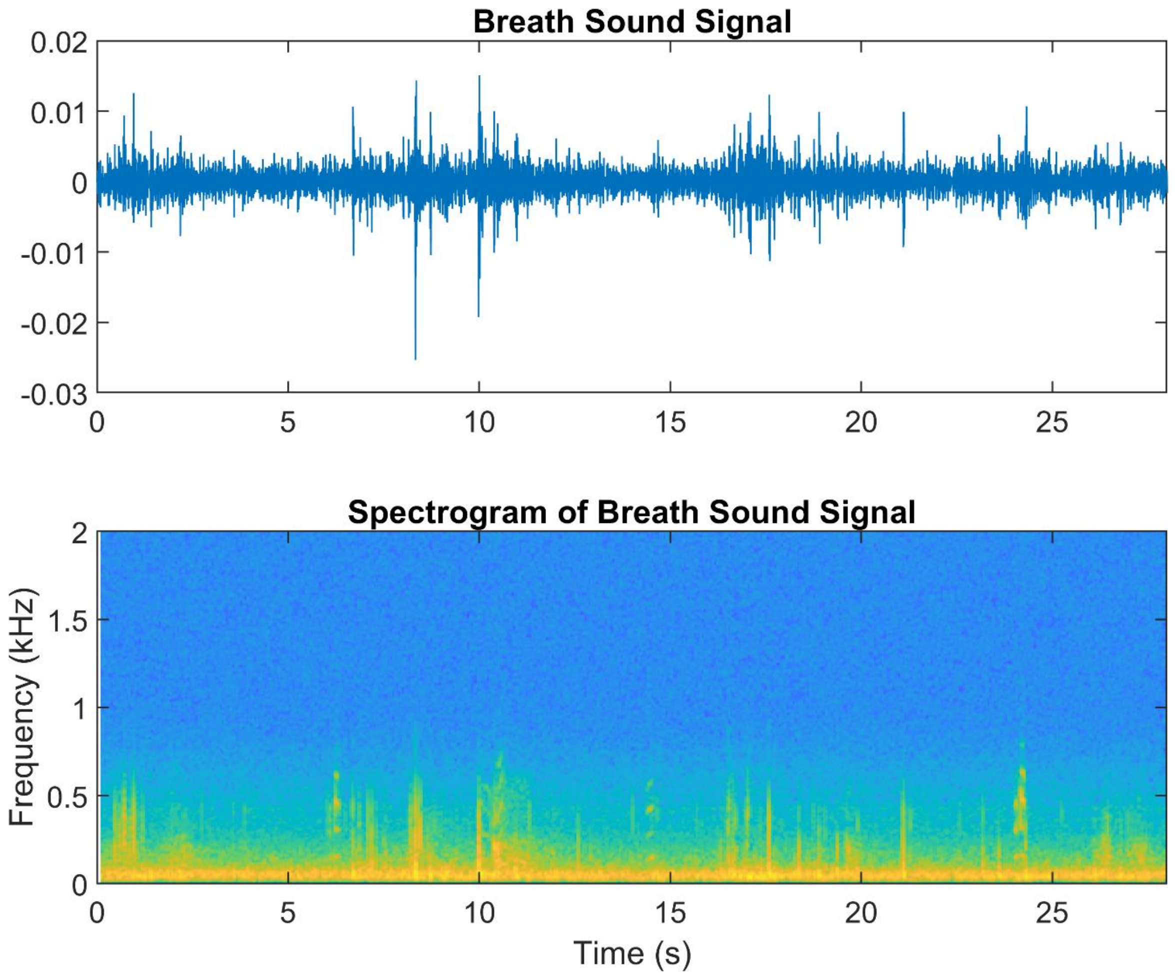

Supplementary Figure 6 | Lung sounds recorded from ADHF patient using Eko stethoscope. High frequency inspiratory crackles recorded from patient with heart failure from the lower lobes on the posterior side indicating accumulation of fluid in the lungs. Important information regarding the breathing patterns is not captured using the Eko stethoscope.

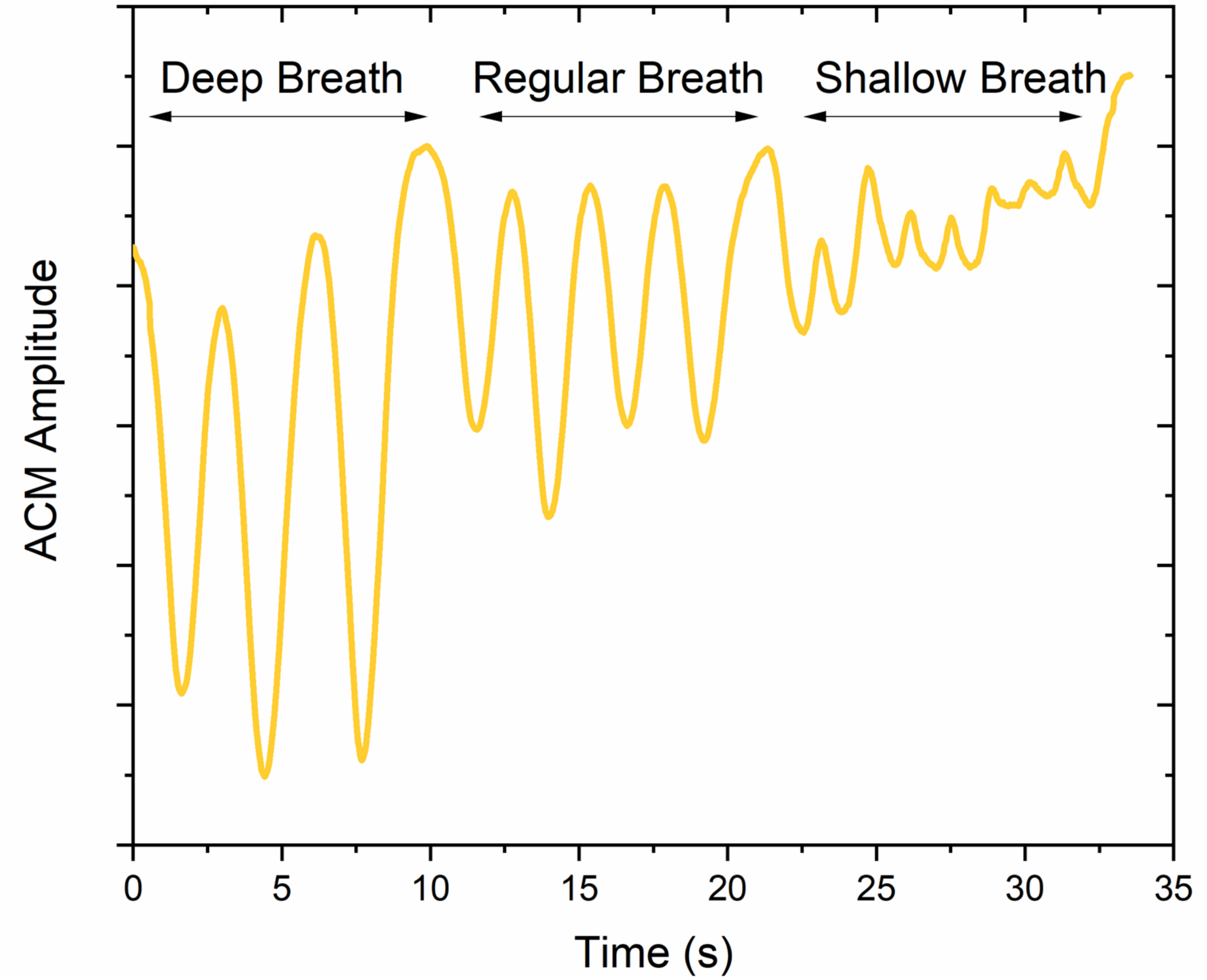

Supplementary Figure 7 | Types of breathing patterns. Low frequency breathing patterns recorded using the ACM sensor. Data is collected from a healthy subject with forced deep breaths, normal breaths and shallow breaths. A test protocol involving 10 seconds of deep breathing followed by 10 seconds of normal breathing and 10 seconds of shallow breathing is followed. The recorded signals only represent expected relative amplitudes and signal patterns between deep, normal, and shallow breathing. The respiratory rate is computed by the periodicity of the breathing pattern waveform.

Supplementary Tables

Supplementary Table 1. Specifications for the Accelerometer Contact Microphone

MEMS Specification	
Sensor Dimension	1.6 mm × 1.6 mm
Package Dimension	2.8 mm × 2.8 mm
Nano-gap Size	250 nm
F_{res}	14.3 kHz
Scale Factor	54.2 fF/g
BNEA	5.3 µg/√Hz
MEMS Device + Interface IC	
Scale Factor	271 mV/g
Operational BW	10 kHz<br>(Limited by electronics)
VRW	55 µg/√Hz
Bias Drift	65 µg
